
JOURNAL INFORMATION
==============================
NLM Title Abbreviation: ScientificWorldJournal
Journal ID: ScientificWorldJournal
NLM Journal ID: 101131163
Journal ID: TSWJ

The Scientific World Journal

ISSN: 2356-6140
EISSN: 1537-744X
Publisher: Wiley

ARTICLE INFORMATION
==============================
PMCID: PMC6009325
PMID: 29973813
DOI: 10.1100/tsw.2002.39
Article ID: 496061
Article version: 1
Subjects: Short Report

The Pedant Human Genome Database ? Functional Annotation of the Human Genome

Schüller Christine M.E. 1
Geier Birgitta 1
Fritz Andreas 1
Salamov Asaf 2
Seledsov Igor 2
Solovyev Victor 2
Frishman Dimitrij 3
Biomax Informatics AG, Lochhamer Straße 11, D-82152 Martinsried, Germany
Softberry Inc., 108 Corporate Park Drive, Suite 120, White Plains, NY, 10604, USA
Institut für Bioinformatik, GSF-Forschungszentrum für Umwelt und Gesundheit, Ingolstädter Landstrasse 1, 85764 Neuherberg, Germany
Publication date: 2002
Electronic publication date: 2002 Feb 5
Volume: 2
First page: 82
Last page: 83
Copyright: Copyright © 2002 Christine M.E. Schüller et al.
Copyright year: 2002
License: This is an open access article distributed under the Creative Commons Attribution License, which permits unrestricted use, distribution, and reproduction in any medium, provided the original work is properly cited.
License URL: https://creativecommons.org/licenses/by/3.0/

==============================
Advances in Gene Technology: The Genome and Beyond –
Structural Biology for Medicine (Proceedings of the 2002 Miami
*Nature Biotechnology* Winter Symposium)
*TheScientificWorld* 2002, 2(S2), 82–83
ISSN 1532-2246; DOI 10.1100/tsw.2002.39

### THE PEDANT HUMAN GENOME DATABASE — FUNCTIONAL ANNOTATION OF THE HUMAN GENOME

Christine M.E. Schüller[1], Birgitta Geier[1], Andreas Fritz[1], Asaf Salamov[2], Igor Seledsov[2], Victor Solovyev[2], and Dimitrij Frishman[3]
[1]Biomax Informatics AG, Lochhamer Straße 11, D-82152 Martinsried, Germany; [2]Softberry Inc., 108 Corporate Park Drive, Suite 120, White Plains, NY, 10604; [3]Institut für Bioinformatik, GSF-Forschungszentrum für Umwelt und Gesundheit, Ingolstädter Landstrasse 1, 85764 Neuherberg, Germany
www.biomax.de; www.softberry.com

**INTRODUCTION**. After the publication of the human genome sequence in February 2001[1], public and private efforts are undertaken to extract valuable information from the wealth of data[2,3]. In our approach we systematically extract all human genes, known and unknown, analyse them in a consistent manner to find out their (possible) function and refine the annotation manually if necessary. All informations are stored in a relational database that can be accessed via a web-based user interface.

**METHOD**. Using the publicly available working draft assembly of the human genome[4], we identified the location of known and putative genes with the exclusive software from Softberry, Inc., Fgenesh++[5]. In the initial analysis, known genes and mRNAs were mapped to the genomic sequence. Subsequently, an *ab initio* gene prediction was performed with the Fgenesh program on the remaining genomic sequences. Predicted proteins were compared to known proteins to refine the gene prediction. The quality of gene identification using this automatic procedure is similar to the quality obtained by manual gene modeling.

The Pedant-Pro Sequence Analysis Suite[6] from Biomax Informatics was used to perform a systematic, comprehensive, and consistent analysis for in-depth functional and structural characterization of the predicted proteome. This characterization includes functional class assignments according to the MIPS functional catalogue as well as assignment of EC numbers, PROSITE patterns, Pfam domains, and SCOP classifications. The software provides DNA and protein viewers for optimized visualization and navigation.

**RESULTS**. The PEDANT Human Genome Database presented here is based on the 12 December 2000 working draft assembly of the human genome and contains over 44,000 genes. Using sequence similarity to the proteomes of *A. thaliana* and *S. cerevisiae* 33.3% of the proteins have been assigned to functional categories (see Fig. 1). In addition to the functional characterization, the Pedant-Pro software performs a series of further functional and structural analyses, like similarity searches, annotation of functional domains, assignment of keywords and EC number, transmembrane domains, etc. Report pages display all the important annotations for each protein, many of which are hyperlinked to the relevant database. Selected information about the proteins is listed in tables. The easy-to-use graphical user interface provides search tools as well as DNA and protein viewers.

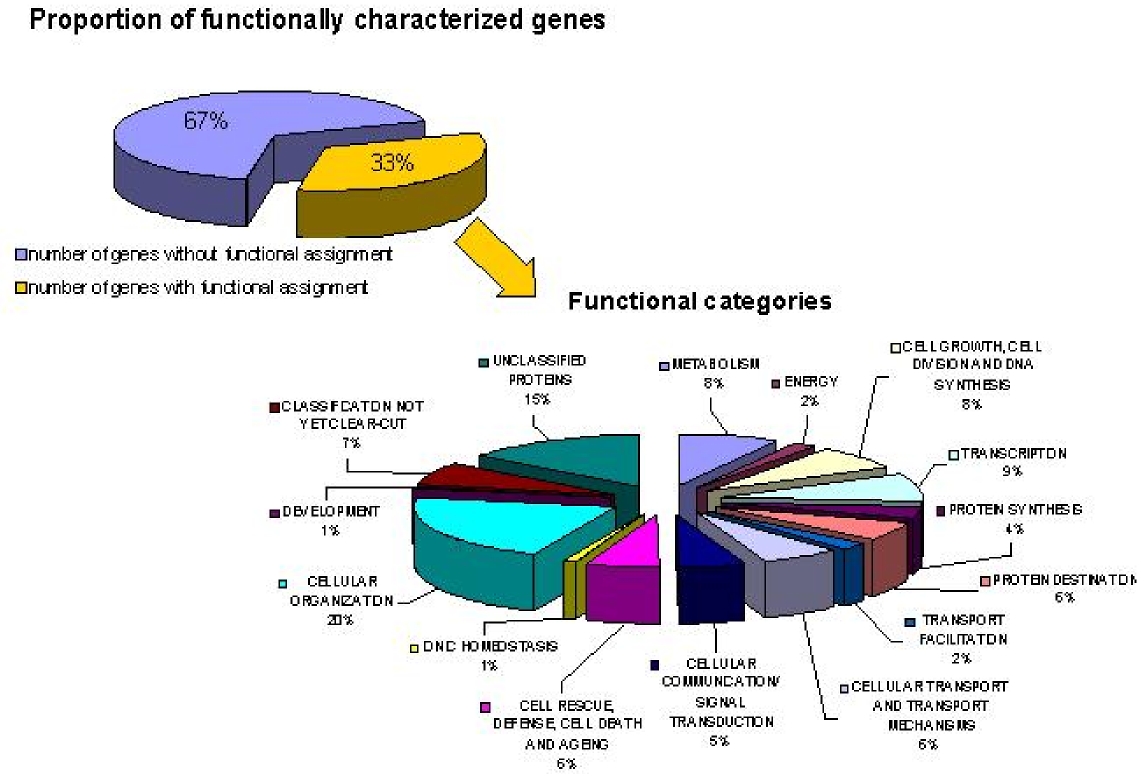

**REFERENCES**

1. The human genome (2001) Nature 409, 745–964.
2. http://www.ncbi.nlm.nih.gov/genome/guide/human/
3. http://www.ensembl.org/
4. http://genome.ucsc.edu/
5. Salamov, A. and Solovyev, V. (1998): http://genomic.sanger.ac.uk
6. Frishman, D. et al (2001) Bioinformatics 17, 44–57.
